# Circle Detection with Adaptive Parameterization: A Bottom-Up Approach

**DOI:** 10.3390/s25082552

**Published:** 2025-04-17

**Authors:** Lin Han, Yan Zhuang, Ke Chen, Yuhua Xie, Guoliang Liao, Guangfu Yin, Jiangli Lin

**Affiliations:** College of Biomedical Engineering, Sichuan University, Chengdu 610065, China; hanl@stu.scu.edu.cn (L.H.); chenke@scu.edu.cn (K.C.); xieyuhua@stu.scu.edu.cn (Y.X.); leosigmoid@stu.scu.edu.cn (G.L.); nic0700@scu.edu.cn (G.Y.)

**Keywords:** adaptive parameter, anti-noise, bottom-up search, circle detection

## Abstract

Circle detection remains a critical yet challenging task in computer vision, particularly under complex imaging conditions where existing measurement methods face persistent challenges in parameter configuration and noise resilience. This paper presents a novel circle detection algorithm based on two perceptually grounded parameters: the perceptual length difference resolution λ, derived from human cognitive models, and the minimum distinguishable distance threshold K, determined through empirical observations. The algorithm implements a local stochastic sampling strategy integrated with a bottom-up circular search mechanism, with all critical parameters in the algorithm derived adaptively based on λ and K, eliminating the need for repetitive hyperparameter search processes. Experiments demonstrate that our methodology achieves an exceptional Fscore of 85.5% on the public circle detection dataset, surpassing state-of-the-art approaches by approximately 7.3%. Notably, the framework maintains robust detection capability (Fscore = 85%) under extreme noise conditions (50% Gaussian noise contamination), maintaining superior performance relative to comparative methods. The adaptive parameterization strategy provides insights for developing vision systems that bridge computational efficiency with human perceptual robustness.

## 1. Introduction

In the field of computer vision, circle detection algorithms play an essential role in diverse areas such as traffic and road safety [1], industrial automation [2,3], medical image processing [4], and scientific research [5]. The accurate detection and precise measurement of circles are essential for numerous vision-based automated tasks.

Many circle detection algorithms utilize the Canny edge detector or its adaptive counterpart to extract image boundaries [6,7]. The Canny edge detector’s reliance is based on carefully selected hyperparameters—Gaussian filter standard deviation (σ) and high/low thresholds (T1, T2) and parameter optimization for specific tasks is often necessary to achieve optimal performance. Guo et al. introduced the bidirectionally connected Canny algorithm to refine edge detection [8], but it does not address the limitations imposed by hyperparameter selection. Yang et al. leveraged Otsu’s method for adaptive threshold calculation in the Canny operator [9], but it lacks sensitivity to subtle nuances and performs poorly in complex images. Meng et al. utilized a locally adaptive Canny algorithm for edge detection [10]; however, this can result in edge discontinuity, potentially compromising the accuracy of detection results. An alternative edge detection algorithm frequently employed is the edge detection parameter-free algorithm (EDPF) [11,12,13,14]. EDPF employs a 5 × 5 Gaussian kernel for image filtering, detects potential edge points based on gradient magnitude and direction, constructs an initial edge map, and finally eliminates spurious edges using the Helmholtz principle [15]. The EDPF demonstrates stronger noise robustness than Canny and operates reliably without hyperparameter tuning. This study employed EDPF as a preliminary edge extractor.

While the circle Hough Transform (CHT) algorithm is used widely in circle detection [16,17,18], it incurs computational and spatial inefficiencies. Furthermore, its accuracy is highly sensitive to numerous hyperparameters, including the accumulator threshold, radius range, accumulator resolution, and voting mechanism design, all of which directly impact result quality [19,20]. Memiş et al. combined the CHT with the integro-differential operator to improve circle matching accuracy [21], but their approach didn’t fundamentally address the high computational cost and hyperparameter dependence inherent in CHT. Kim et al. proposed a two-stage Hough transform algorithm that first uses a 2D accumulator matrix to locate circle centers and then a 1D histogram to determine the radius [22]. This method reduces computational time and memory consumption, but challenge of hyperparameter selection remains.

The random sample consensus (RANSAC)-based algorithm [23,24,25,26] demonstrates heightened efficiency on conventional imagery; its performance wanes when confronted with noisy interference or complex scenes. Moreover, several pivotal hyperparameters inherent in the algorithm, including sampling-point counts, accumulation thresholds, fitting precision requirements, and iteration count, significantly influence the algorithm’s final outputs [27].

To enhance circle detection accuracy, many algorithms have been investigated. Jiang et al. [28] implemented a refined RANSAC approach, augmenting the sampling density within a restricted region to achieve more precise circle parameter estimations. Zhao et al. [14] proposed a method that optimizes candidate circle parameters by verifying inscribed triangles. This method aims to connect as many circular arc segments as possible into a whole. Other researchers have investigated diverse optimization algorithms to further elevate the accuracy of circle parameter estimation [29,30,31]. These algorithms often introduced additional hyperparameters, compounding the challenges of hyperparameter optimization. Moreover, these approaches employed a greedy strategy to assemble circular arcs, thereby reducing the vast search space, yet they are prone to falling into the trap of local optima.

Researchers have also applied deep learning to circle detection tasks. Mekhalfi et al. compared the performance of three deep learning models (YOLOv5, Transformer, and EfficientDet) in detecting circular agricultural patterns in desert regions [32]. The authors constructed two datasets and evaluated the models’ respective performances on them. Yue et al. employed an enhanced YOLO algorithm for dish detection [33], which included circular object recognition. Gai et al. proposed a cherry detection algorithm based on the YOLO framework [34], replacing traditional anchor boxes with circular bounding boxes to better match the fruit’s morphology, thereby improving detection accuracy. Similarly, Liu et al. introduced the YOLO-Tomato model [35], utilizing circular bounding boxes (C-Bboxes) that better conform to tomato shapes to enhance both detection precision and IoU calculation accuracy in non-maximum suppression. These approaches predominantly employ deep localization models to identify circular objects in images, yielding promising results. These approaches are tailored specifically for detecting circular objects in particular contexts, rather than serving as general-purpose circle detection algorithms. Moreover, these methods generate bounding boxes other than precise circular parameters. In scenarios demanding accurate geometric measurements, traditional circle detection algorithms remain indispensable for precisely determining the parameters of circles within these bounding regions.

This study leveraged the EDPF algorithm for preliminary edge detection. Subsequently, a novel patch-based random sample consensus algorithm (pRC) was employed to detect linear segments and circular arcs in images, and a hierarchical search algorithm (HS) was proposed to combine these geometric primitives to candidate circles. Based upon these candidates, we introduced a circle parameter synthesis algorithm (CPS) to search, aggregate, and refine circular parameters. Finally, the arc length ratio was calculated to filter spurious detections while preserving true circle parameters.

The pRC algorithm partitions the entire image into patches and applies the RANSAC process within each patch to extract primitive geometric structures. This divide-and-conquer approach effectively addresses the hyperparameter selection challenges commonly encountered in traditional RANSAC algorithms. Our HS algorithm employs a bottom-up search strategy to iteratively coalesce circular arcs, ultimately converging to the globally optimal combination solution. The CPS searches for arc segments with similar geometric parameters, systematically aggregates them through a bottom-up hierarchical way, and thereby identifies optimal circular parameter configurations. Starting from edge points, these three algorithms iteratively aggregate candidate circle parameters to produce reliable circle estimations. The incorporation of a search strategy makes each step achieve a globally optimal solution. In contrast to other algorithms that employ a greedy strategy that often leads to local optima, our methodology exhibits enhanced robustness against noise.

## 2. Methodology

**Figure 1:**
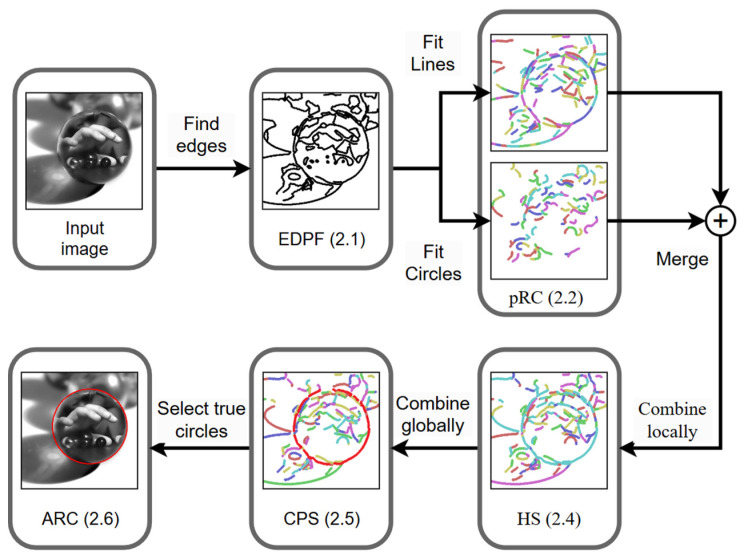
The flowchart of the algorithm, distinct colors denote different segments.

The flowchart of the algorithm is illustrated in Figure 1. For the input image, we first employed the EDPF to extract edge points. Subsequently, the pRC algorithm was applied to extract line segments and circular arcs as preliminary structural elements. The HS algorithm was then utilized to integrate adjacent structural elements into candidate circles. This was followed by the CPS to globally combine and optimize circle parameters. Finally, true circle parameters were obtained through arc length ratio criteria (ARC).

### 2.1. Edge Point Detection

This work leveraged the EDPF algorithm for edge point detection; subsequently, a thinning algorithm [36] was applied to ensure the detected edges had a consistent width of one pixel. The EDPF filters the image with a 5 × 5 Gaussian kernel, identifies pixels with significant gradient magnitudes and directions as potential edge points, then connects them to form an initial edge map, and finally removes spurious edges using the Helmholtz principle [15]. Compared to the Canny algorithm, EDPF exhibits enhanced noise robustness, delivers connected edges other than discrete points, and, as its name suggests, performs reliably without hyperparameter tuning.

### 2.2. The pRC Algorithm

The pRC is a modified RANSAC approach to uncover preliminary structures within images. The image is partitioned into patches (see Section 2.2.1), and an optimized RANSAC was applied within each patch to detect structural elements (see Section 2.2.2). Then non-maximum suppression was utilized to eliminate redundant detection results (see Section 2.2.3). Since circular arcs with larger radii may approximate straight line segments within the local patches, both line segments and arc detection are required. This patch-based strategy enables parallel processing to increase computational speed and facilitates adaptive parameter estimation, thereby enhancing versatility across diverse images.

#### 2.2.1. Edge Points Partitioning

We segmented the input image into a series of L×L square patches (L=32) with a stride of S=16, and edge points were extracted within each patch. Patch size and stride remain invariant regardless of the image dimensions, and patches along image edges lacking sufficient pixels were discarded. This configuration resulted in a 50% overlap between adjacent patches, which guaranteed that segments traversing patch boundaries also be detected. Values for L and S were derived from K, as detailed in Appendix A.

#### 2.2.2. RANSAC on Patches

RANSAC was then applied to each patch to identify line segments and circular arcs, with the critical parameter—iteration count N—calculated adaptively. Here, we define $p_{f}=0.99$ as the desired probability of detecting the geometric structure, $m_{min}=16$ as the minimum number of points required to form a structure, and V as the set of all edge points in the patch. The problem can be posed as a probabilistic proposition: “Given a set of edge points V, how many repetitions (N) are required to ensure a probability $p_{f}$ for structures with length no less than $m_{min}$”. Therefore, N can be calculated using Equations (1) and (2).

(1) $$b=1-{\left(\frac{m_{min}}{\left|V\right|}\right)}^{t}$$

(2) $$N=\left\lceil \log_{b}\left(1-p_{f}\right)\right\rceil$$

where |·| denotes the number of elements in a set, and t represents size of support points for a structural entity. For a line segment, t=2; for a circle, t=3. The notation $\left\lceil \cdot \right\rceil$ indicates the ceiling function, which rounds a number up to the nearest integer. The calculation for $m_{min}$ was detailed in Appendix A.

To maximize structure detection, we optimized RANSAC’s random sampling process. The algorithm employs an iterative strategy for structural extraction. During each iteration, it performs N random samplings and parameter estimations. At each step, t support points were randomly selected from V, and structural parameters were estimated using the least squares method. Subsequently, the algorithm identified the subset of points in V that satisfy the distance collinearity constraints relative to the derived parameters. When the number of identified collinear points exceeds the predefined threshold $m_{min}$, the current parameters and their corresponding point set are recorded. Upon completing N cycles, the algorithm selects the optimal parameters from all recorded instances for the output result, simultaneously removing the corresponding points from V. This process repeats until no qualifying point set can be found or the remaining points in V fall below the threshold $m_{min}$ (see Algorithm 1).
**Algorithm 1:** The pRC algorithm
Input: V: All edge points in patch
Output: R←∅1Calculate the loop count N using (1–2)2D←∅3REPEAT N times:4   $p_{s}\leftarrow \left\{p_{1}\ldots p_{t}\right\}\;where\;p_{1}\ldots p_{t}\in V$//for line segments, t = 2; for circular arcs, t = 35   $If\;\exists p_{a},p_{b}\in p_{s},\left\|p_{a},p_{b}\right\|<K$, THEN CONTINUE6   
M←Calculate structure parameters
7   
$d_{n}\leftarrow \{P_{s}|\forall P_{s}\in V,dist(P_{s},M)<TD_{P}\}$
8   
$IF\;\left|d_{n}\right|\geq m_{min}$ $\mathrm{THEN}\;D\leftarrow D\cup \{d_{n}\}$
9End REPEAT10IF D=∅ THEN GOTO line 1511$S\leftarrow \underset{d\in D}{\mathrm{arg max}}\left|d\right|$12V←V−S13R←R∪{S}14$IF\;\left|V\right|>m_{min}$ THEN GOTO line 115RETURN R

Points were considered on the arc if their distance to the curve is less than one pixel; therefore, the $TD_{P}$ in line 7 is set to 1 pixel. The empirical hyperparameter K was set to 3 pixels, which represents the minimal perceptible difference, detailed in Appendix A.

#### 2.2.3. Non-Maximum Suppression (NMS)

Since each patch overlaps with its adjacent patches, redundant detection of structural elements may occur. It is essential to utilize a non-maximum suppression algorithm to eliminate superfluous detection results for both line segments and circular arcs. Let $R_{0}=\{s_{1},s_{2},\ldots ,s_{n}\}$ denote the initial set of all segments, and $R^{*}$ be the result of NMS, the algorithm iteratively selects the longest segment and extirpates any structures exhibiting overlap exceeding K. This process repeats until all results have been processed, see Equations (3)–(5), as follow:

(3) $$S_{t}^{*}=\underset{S\in R_{t}}{\mathrm{argmax}}\left(\left|S\right|\right)$$

(4) $$R_{t+1}=\left\{S\in R_{t},\left|{S-S}_{t}^{*}\right|>K\right\}$$

(5) $$R^{*}=\underset{t}{⋃}\left\{S_{t}^{*}\right\}\;until\;R_{t+1}=\emptyset .$$

where S denotes the set of points lying on the line, $B-A=\left\{x|x\in B,x\notin A\right\}$ denotes the set difference of B and A, and |·| indicates the cardinality of the set.

### 2.3. Distance Metric

We employed distance metric to evaluate the error of the circle parameters fitted by the least square. Given a segment $S=\{(x_{1},y_{1}),(x_{2},y_{2})\ldots (x_{n},y_{n})\}$, the distance from S to the circle (characterized by parameters a, b, and r) is defined as the maximum distance from all points on this line segment to the circle, as shown in (6).

(6) $$D=\underset{1\leq i\leq n}{\max}\left\{\left|\sqrt{{\left(x_{i}-a\right)}^{2}+{\left(y_{i}-b\right)}^{2}}-r\right|\right\}$$

If D is less than the distance-limit value $TD_{S}$, the line segment was considered to meet the distance criterion, and $TD_{S}$ was calculated using (7), as follows:

(7) $$TD_{S}=\left\{\begin{array}{c} TD_{p}\;\;\;\;\;\;\;\;\;\;\;\;\;\;\;\;\mathrm{if}\;r*\lambda <TD_{P}\\ K\;\;\;\;\;\;\;\;\;\;\;\;\;\;\;\;\;\;\;\;\;\mathrm{if}\;r*\lambda >K\;\;\;\;\;\\ r*\lambda \;\;\;\;\;\;\;\;\;\;\;\;\;\;\;\;\;\mathrm{otherwise}\;\;\;\;\;\;\;\;\; \end{array}\right.$$

where λ = 0.05 was another hyperparameter that represented the minimum length difference discrimination ratio between line segments (see Appendix A).

### 2.4. The HS Algorithm

After obtaining the point sets of line segments and arc segments (collectively termed “segments”), we added all segments to the segment library (denoted as $R^{*}$). We employed a bottom-up hierarchical search algorithm (HS) method to incrementally construct candidate arcs and their parameters. The algorithm starts by employing least-squares to fit circular parameters for each segment. Subsequently, it iteratively identifies and merges the most suitable adjacent segments. This process continues until no further advantageous mergers are possible; see Algorithm 2.
**Algorithm 2:** Calculate candidate circle parameters 
Input: $R^{*}$: All segments derived from section C.
Output: $L_{C}$1$L_{C}\leftarrow \left\{\langle Seg,P\rangle |\forall Seg\in S_{p},P=LSQ_{V}(Seg)\right\}$ //$LSQ_{v}$: A function that fits circular parameters via least squares, returns parameters if its distance criterion is met, see Equations (6) and (7); otherwise, returns None.2dirty←false3$FOR\;EACH\;\langle SegA,P\rangle \;in\;L_{C}:$4   
$Ext\leftarrow Search\text{\_}Extension\left(SegA,L_{C}\right)$
5   
$SegB,E_{min}\leftarrow None,\infty$
6   
FOR EACH S in Ext:
7      Q←S+SegA8      
$\langle a,b,r\rangle \leftarrow LSQ(Q)$9      
$D\leftarrow \underset{\langle x_{i},y_{i}\rangle \in Q}{\max}\left\{\left|\sqrt{{\left(x_{i}-a\right)}^{2}+{\left(y_{i}-b\right)}^{2}}-r\right|\right\}$
10      $\mathrm{IF}\;D<E_{min}$ AND D< $TD_{S}$ $\mathrm{THEN}\;SegB,E_{min}\leftarrow S,D$11   End FOR EACH12   IF SegB≠None THEN:13      
SegC←SegA+SegB
14      
$L_{C}\leftarrow \left(L_{C}-\left\{SegA,SegB\right\}\right)\cup \left\{\langle SegC,LSQ_{V}(SegC)\rangle \right\}$
15      
dirty←true
16   ELSE IF P=None THEN: $L_{C}\leftarrow L_{C}-\left\{SegA\right\}$17End FOR EACH 18IF dirty=true THEN: 19   
$L_{C}\leftarrow NMS(L_{C})$
20   GOTO line 2 21End IF22$\mathrm{RETURN}\;L_{C}$

**Figure 2:**
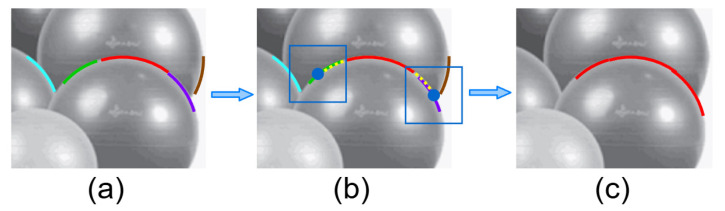
Schematic representation of extended segment search, using circular arcs as an illustrative example. Various colors signify distinct arcs, while dashed lines denote outward-expanding trajectories, and the blue bounding box delineates the search area, with blue dots marking extension points. Arrows illustrate the algorithmic flow.

In line 4, the $Search\text{\_}Extension(SegA,L_{C})$ function was implemented to identify potential candidate segments for combination by examining square regions that extended beyond both endpoints of SegA within $L_{C}$. The square regions possess a fixed side length of $m_{min}$, with their positions determined through the following protocol: for segments unbounded with circular parameters, linear extension was implemented by extending outward along the linear trajectory by $m_{min}/2$. For circular-parameter-bounded segments, extension was performed along the arc length by $m_{min}/2$. Segments were identified as candidates when any of their constituent points fell within the corresponding square region. The illustration is shown in Figure 2a image with the identified arcs; Figure 2b extend the central arc (red) with its parameter in both directions by a distance of $m_{min}/2$ (yellow dashed lines). The extension points (blue dots) are centers for square search regions (blue squares) of side length $m_{min}$. Candidate segments are found within these regions (cyan and green on the left; purple and brown on the right); Figure 2c finally, an optimal combination pattern is sought, merging relevant segments into a unified arc (red, green, and purple combined).

### 2.5. The CPS Algorithm

In Section 2.4, we obtained candidate circle parameters along with their corresponding point sets. These parameters were collectively referred to as the circle parameter library $L_{C}=\left\{\langle S_{1},M_{1}\rangle ,\langle S_{2}.M_{2}\rangle ,\ldots ,\langle S_{n},M_{n}\rangle \right\}$, where $S_{i}$ represents the edge points of ith circle, $M_{i}=\langle a_{i},b_{i},r_{i}\rangle$ represents the ith circle’s parameters: center coordinates $\left(a_{i},b_{i}\right)$ and radius $r_{i}$. We introduced the circle parameter synthesis (CPS) algorithm to search, aggregate, and refine circular parameters. For each circle in $L_{C}$, the algorithm identifies the five nearest candidate circles within the 3D parameter space, merges their arc points to form potential new circles, and selects the optimal combination based on minimum distance. This procedure repeats until convergence, outputting the refined parameter collection, see Algorithm 3. To accelerate analogous circle retrieval, we employed the kd-tree algorithm [37].
**Algorithm 3:** Circle Space Refinement
Input: $L_{C}$: Circle parameter library
Output: $L_{R}:$ Refined Circles1$L_{R},dirty\leftarrow \emptyset ,false$2FOR EACH $\langle S_{A},M_{A}\rangle$ in $L_{C}$:3   
$C_{S}=\underset{\langle S_{B},M_{B}\rangle \in L_{C},S_{B}\neq S_{A}}{\mathrm{arg mi}n_{5}}\left\|M_{A},M_{B}\right\|$
4   
$C_{G}=\left\{\langle S_{C},M_{C}\rangle |S_{C}=S_{A}+S_{B},M_{C}=LSQ_{V}(S_{C})\right\}$
5   $S_{G}=\underset{\langle S,M\rangle \in C_{G}}{\mathrm{arg min}}dist(S,M)$   //Select the circle minimizing the distance metric defined in Equation (6).6   IF $S_{G}=None$ THEN CONTINUE 7   
dirty←true
8   
$L_{R}\leftarrow L_{R}\cup \{S_{G}\}$
9END FOR EACH 10$L_{R}\leftarrow NMS(L_{R})$11IF dirty=true THEN 12   
$L_{C}\leftarrow L_{R}$
13   GOTO line 1 14END IF 15RETURN $L_{R}$

### 2.6. Arc Length Ratio Criteria

After obtaining the circle parameters $\langle S_{n},M_{n}\rangle \in L_{R}$, $M_{n}=\langle a_{n},b_{n},r_{n}\rangle$, $S_{n}=\left\{\langle x_{n}^{1},y_{n}^{1}\rangle ,\langle x_{n}^{2},y_{n}^{2}\rangle ,\ldots \langle x_{n}^{K},y_{n}^{K}\rangle \right\}$, $a_{n}$, $b_{n}$, and $r_{n}$ respectively represent the X-coordinate, Y-coordinate of the n-th circle’s center, and its radius, while $x_{n}^{i}$ and $y_{n}^{i}$ denote the coordinates of the n-th point on the n-th circle. The arc completion was performed to select optimal circle parameters using Equations (8) and (9), as follow:

(8) $$TL_{n}=\max\left(0.9-\left(0.9-\alpha \right)*\frac{r_{n}}{K/\lambda },\alpha \right)$$

(9) $$C_{T}=\left\{M_{n}|\langle S_{n},M_{n}\rangle \in L_{R},\frac{\left|S_{n}\right|}{2*\pi *r_{n}*\eta }>TL_{n}\right\}$$

where 1≤n≤N, N denotes the total number of circles detected in Section 2.5. The empirical parameter α=0.3 represents the minimum arc completion threshold. $TL_{n}$ indicates the completion threshold for circle n, where arcs exceeding $TL_{n}$ are considered valid circles. The value of $TL_{n}$ ranges from α to 0.9 according to circle radius variations, with smaller radii requiring higher completion levels. As demonstrated in Appendix A, η = 0.9 denotes the ratio between ideal and digitized circle circumferences. $C_{T}$ represents the parameters of finally detected circles.

## 3. Experiments and Results

### 3.1. Datasets

We employed the dataset collected by Zhao et al. [14], which comprises four distinct components:

- Dataset-Mini: A benchmark test set containing 10 low-resolution images with fixed pixel dimensions, presenting spectral reflection and occlusion challenges, originally established by Akinlar and Topal [11].
- Dataset-GH: A complex real-world collection of 258 grayscale images from diverse environments, exhibiting blurred boundaries, partial occlusions, and substantial radius variations.
- Dataset-PCB: An industrial dataset comprising 100 printed circuit board (PCB) images with concentric circular structures, containing significant noise pollution and edge blurring that present detection challenges.
- Dataset-MY: A newly compiled dataset of 111 smartphone-captured images featuring multiple circular targets per frame, characterized by cluttered backgrounds, perspective distortions, and elliptical deformations caused by angular perspectives.

The datasets above were widely used in circle detection tasks [6,7,12], and all images were manually annotated with ground truth.

### 3.2. Environment

The testing hardware environment comprised an Intel(R) Core (TM) i7-10700 CPU @ 2.90 GHz with 16-core architecture and 32 GB RAM. The software environment utilized Ubuntu 22.04.4 LTS, with the algorithm implementation coded in Rust programming language, employing the rayon crate for parallel processing during pRC, HS, and CPS.

### 3.3. Comparison Metrics

In this study, we integrated the four data sets and calculated the comprehensive algorithm performance across all images. We employed Precision, Recall, and Fscore metrics to evaluate algorithmic performance, as defined in Equations (10)–(12).

(10) $$Precision=\frac{TP}{TP+FP}$$

(11) $$Recall=\frac{TP}{TP+FN}$$

(12) $$Fscore=2*\frac{Precision*Recall}{Precision+Recall}$$

A detected circular parameter $M_{n}$ was classified as a true positive (TP) if its intersection-over-union ratio (IoU) with any ground truth label $L_{i}$ exceeded 0.8; otherwise, it was designated as a false positive (FP). The IoU threshold (0.8) was adopted in many circle detection tasks [6,7,14]. Ground truth labels lacking corresponding detected parameters were identified as false negatives (FN).

(13) $$IoU=\frac{area\left(M_{n}\right)\cap area\left(L_{i}\right)}{area\left(M_{n}\right)\cup area\left(L_{i}\right)}$$

### 3.4. Performance

**Figure 3:**
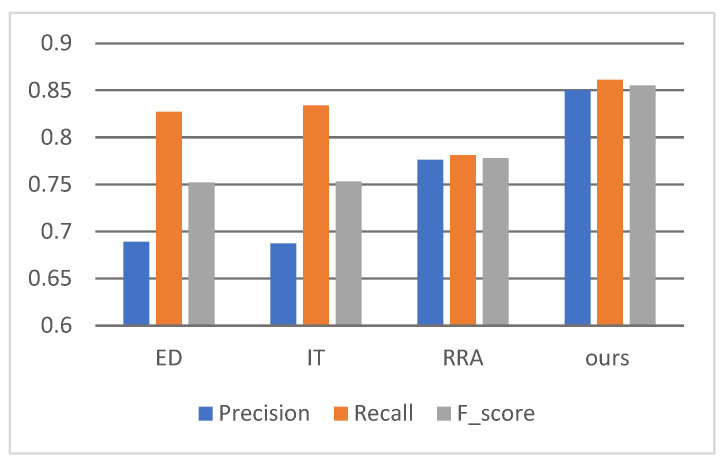
The performance of all algorithms using a bar chart.

To evaluate our model’s performance, we conducted comparative evaluations against three established circle detection methods: the EDCircle algorithm (ED) [12], the inscribed triangles method (IT) [14], and the regionalized radius assistance technique (RRA) [7]. Their source codes are publicly available online, with ED and IT implemented in C++, while RRA was developed using MATLAB. The C++ code was compiled using GCC version 11.4.0, while the MATLAB scripts were executed on MATLAB 2018b. All comparative methods had demonstrated superior performance in prior circle detection research, and the hyperparameters of each algorithm were set to the optimal values as specified in their respective original papers. The performance of each algorithm is summarized in Table 1. We analyzed the experimental results and plotted them in a bar chart (Figure 3). The results demonstrate that our algorithm outperformed the comparative methods across all performance metrics, surpassing the second-best algorithm by 7.2% in Fscore. Furthermore, our approach exhibited greater consistency across all three metrics compared to other methods, suggesting enhanced result uniformity and reduced sensitivity to data distribution variations. This can improve consistency and enhance the algorithm’s applicability across diverse practical scenarios. Table 1’s fifth column presents the average execution times of algorithms across all images. Evidently, our algorithm requires more computation time than the real-time circle detection methods ED and IT. Nevertheless, with an average runtime under 0.8 s, it remains well within practical usability thresholds.

**Figure 4:**
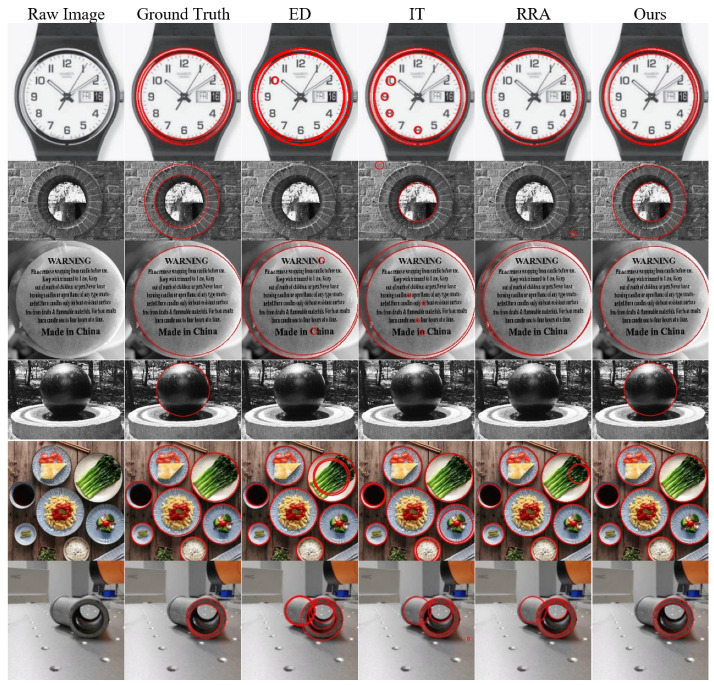
The circle detection samples, arranged from left to right as follows: the raw image, ground truth, and the detection results of ED, IT, RRA, and ours. All algorithms exhibited some false or missed detections, but our method performed better overall. The red circles shown in the figures indicate the circular shapes detected by the algorithms.

Representative experimental results are visualized in Figure 4. ED exhibits lower detection rates on some images while producing a higher number of false positives in others. IT generally presents more false positive detections. RRA shows balanced performance but yields inferior results compared to our method.

### 3.5. Noise Test

**Figure 5:**
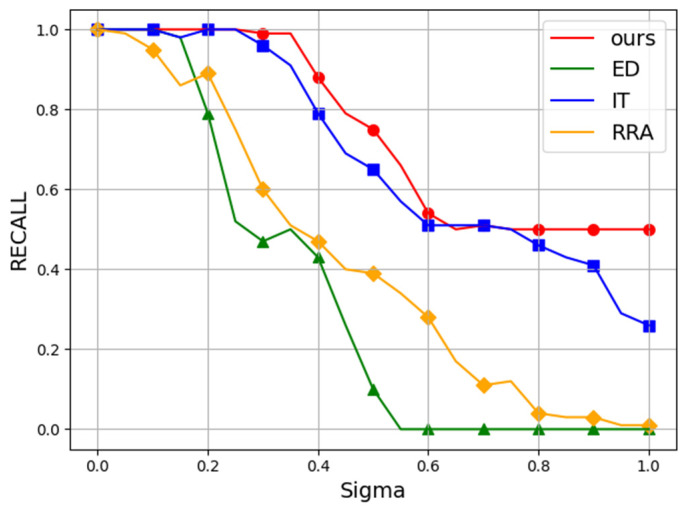
The Recall performance of all algorithms under noise interference. Red, green, blue, and yellow represent our method, ED, IT, and RRA, respectively. The vertical axis represents Recall, and the horizontal axis denotes the standard deviation (σ) of the applied Gaussian noise, ranging from 0% to 100%.

**Figure 6:**
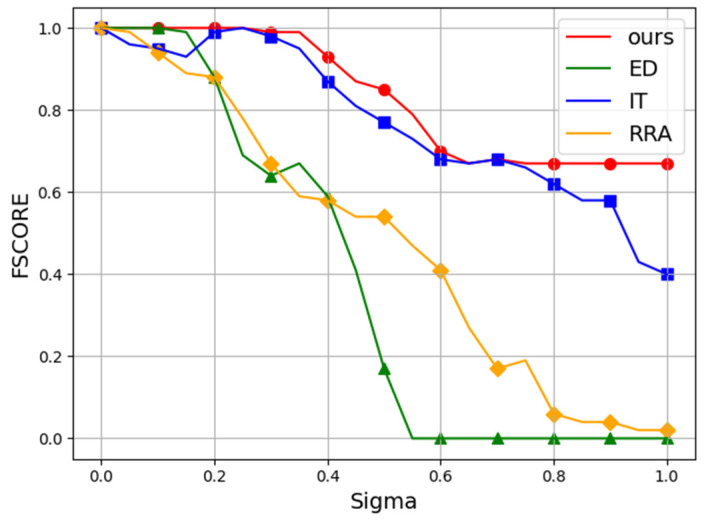
The Fscore performance of all algorithms under noise interference. The vertical axis represents Fscore, and the horizontal axis denotes the standard deviation (σ) of the applied Gaussian noise, ranging from 0% to 100%.

**Figure 7:**
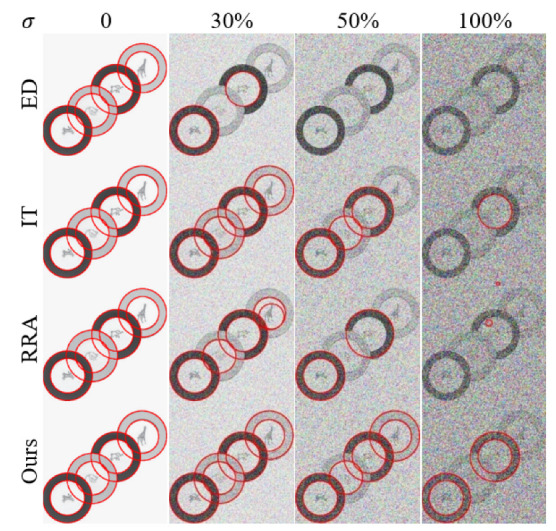
The outcomes of each algorithm on images with increasing noise (σ = 0–100%). Each row represents a different algorithm, while columns indicate noise levels. The red circles shown in the figures indicate the circular shapes detected by the algorithms.

In addition, we evaluated the robustness of each algorithm against Gaussian noise perturbations. Following conventional practice, we employed the 400 × 300 “plates” image from Dataset Mini. Starting with a Gaussian noise level of σ=0, we progressively increased the noise intensity in 5% increments until reaching σ=100%. The Gaussian noise was added to the original image, with pixel values subsequently clipped to [0,1]. Experiments were repeated 10 times at each noise level, averaging the Recall and Fscore results. The average results are presented as line charts in Figure 5 and Figure 6, whereas Figure 7 shows representative detection outputs from the algorithms under different noise conditions.

It can be seen in Figure 7 that our algorithm demonstrated robustness by consistently avoiding false positives, even under high noise conditions, where some other algorithms falter. In Table 2 and Table 3, the Recall and Fscore of each model across various noise levels are presented. Our method surpasses competing algorithms, attaining an impressive Fscore of 0.85 even amidst 50% noise interference.

## 4. Conclusions

This study proposes an automatic circle detection framework that requires no manual parameter tuning. The algorithm implemented efficient detection through a four-stage process based on the divide-and-conquer principle and bottom-up search: First, the EDPF algorithm extracted continuous boundary points, eliminating reliance on Gaussian filtering and thresholds in Canny operator. Second, pRC divided images into local areas for adaptive detection of line segments and arcs. Third, HS and CPS dynamically merged adjacent geometric structures to approximate true circle parameters. Finally, the arc length ratio criteria filtered pseudo-circles. The innovation manifested in three aspects: (1) a region-based local parameter adaptation mechanism converted global hyperparameters into dynamic regional computations, eliminating parameter tuning burdens in complex scenarios; (2) intuitive parameter design aligned with human cognitive patterns enhanced algorithmic transparency; and (3) geometry-constrained hierarchical structure aggregation effectively resolved arc discontinuity caused by occlusion. Experiments demonstrated an 85.5% Fscore on industrial and natural scene datasets, achieving a 7.3% improvement over suboptimal methods. The framework’s robustness also outperformed existing algorithms due to parameter adaptation and hierarchical optimization. Its core design could also be extended to complex tasks like ellipse detection, providing high-generalization solutions for industrial quality inspection and medical image analysis.

## Figures and Tables

**Table 1:** The performance comparison of the algorithms.

Methods	Precision	Recall	Fscore	Time/s
ED [12]	0.689	0.827	0.752	0.023
IT [14]	0.687	0.834	0.753	0.12
RRA [7]	0.776	0.781	0.778	6.1
Ours	0.850	0.861	0.855	0.77

**Table 2:** The Recall score of all methods on Gaussian noise perturbations.

σ	RRA	ED	IT	Ours
0%	1	1	1	1
30%	0.6	0.47	0.96	0.99
50%	0.39	0.1	0.65	0.75
100%	0.01	0	0.26	0.5

**Table 3:** The Fscore of all methods on Gaussian noise perturbations.

σ	RRA	ED	IT	Ours
0%	1	1	1	1
30%	0.67	0.64	0.98	0.99
50%	0.54	0.17	0.77	0.85
100%	0.02	0	0.4	0.67

**Figure A1:**
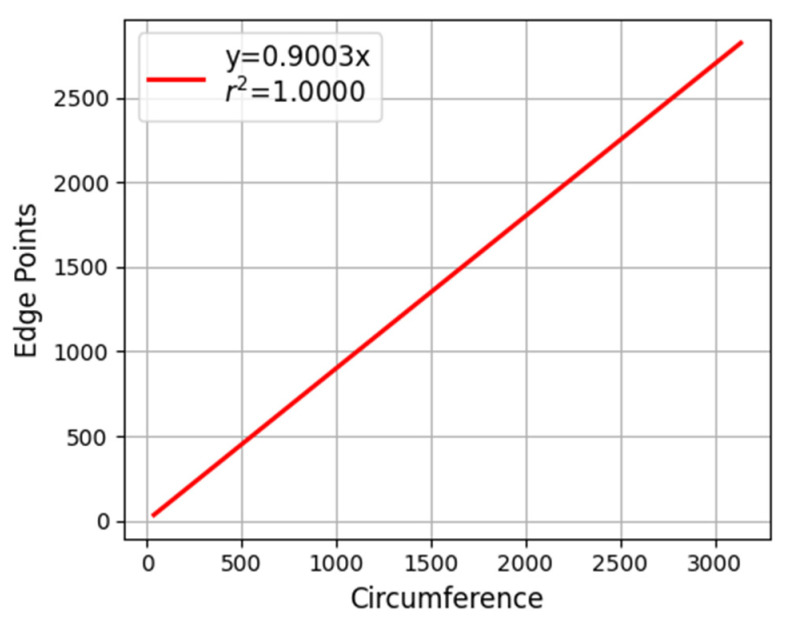
The linear relationship between the theoretical circumference of a circle at different radii and the number of its boundary points.

## Data Availability

The data used in this study was obtained from the GitHub repository at https://github.com/zikai1/CircleDetection, accessed on 30 November 2024.

## Abbreviations

The following abbreviations are used in this manuscript:

EDPF	Edge detection parameter-free algorithm
CHT	Circle Hough Transform
RANSAC	Random sample consensus
pRC	Patch-based random sample consensus algorithm
HS	Hierarchical search algorithm
CPS	Circle parameter synthesis algorithm

## Appendix A. Parameters and Their Determination Methodologies

The parameters employed throughout this study, their corresponding assigned values, and explanatory rationales are meticulously documented in Table A1:

**Table A1:** Parameters, their values, and explanations.

Parameter	Value	Explanation
K	3	Minimum perceptible distance discernible by human sensory capabilities.
λ	0.05	Minimum length difference discrimination ratio between line segments.
α	0.3	An empirical parameter, employed to ascertain the minimal arc completion of an authentic circle.
η	0.9	Ratio between ideal and digitized circle circumferences.
$m_{min}$	16	Minimum length of a valid line segment.
L	32	Length of the side of the square area employed to identify local segments.
S	16	Sliding step length of the square region utilized for detecting local segments.

1. Value of K

K represents the minimum distinguishable pixel distance. Pixels separated by less than this threshold are considered perceptually connected. The actual value depends on the angular resolution of human vision, viewing distance, and display resolution parameters, making theoretical determination prohibitively challenging. In this study, we referred to similar research works [38,39] and assigned K a value of 3 pixels.

2. Value of λ

The parameter λ represents the discrimination threshold for lengths of two non-adjacent line segments, which corresponds to the Weber’s fraction for length perception. Ono’s experimental findings [40] demonstrated that under non-simultaneous viewing conditions, the human eye’s response threshold to length stimuli varied between 2.16% and 5.12% across different experimental configurations. Subsequent research by Wang et al. [41] established a discrimination threshold range of 3.10% to 5.18% for object size variation. In this study, we assigned λ = 0.05 to accommodate broader application scenarios.

3. Value of α

The parameter α was the arc completion parameter used during the screening stage to identify true circles. During the circle parameter fitting stage, some local noise instances might be incorrectly combined into circular shapes, though such combinations generally did not form extensive arcs. An appropriately chosen α could effectively filter these false positives while preserving valid detections. However, excessively large α values might eliminate genuine circles due to incomplete arc detection or occlusion scenarios. Through empirical observation, we found that when an appropriately sized arc exhibited over 30% completion, humans consistently perceived it as a complete circle. Based on this finding, we set α to 0.3 in our implementation.

4. Value of η

Mathematically, for a circle of radius r, the circumference equals 2πr. In digital image processing, however, the focus shifts to quantifying boundary pixels of digitally represented circles. To investigate the relationship between boundary pixel counts and theoretical circumference values, we employed the Bresenham algorithm [42] to generate circles with radii ranging from 6 to 500 pixels. The boundary pixels were counted and subsequently compared against corresponding theoretical circumference values. The experimental data were shown in Figure A1. The ratio of the boundary pixel counts to the theoretical circumference (denoted as η) is approximately equal to 0.9, and there is a strong linear correlation between the two.

5. Value of $m_{min}$, L, and S

Based on the definition of K above, we assume that the radius of the smallest detectable circle is K, yielding the length of the smallest circle’s boundary as 2πKη≈16.96. For the convenience of calculation, we rounded down $m_{min}$ to 16, and this result coincides with the research of Jia et al. [43] and Zhao et al. [14]. Given $m_{min}$, we can determine an appropriate patch size. For a circle with a large radius, its arc approximates a straight line within the patch. Ideally, to ensure detection of all arc points, a patch size equal to $m_{min}$ is sufficient; however, since the extracted boundaries are often discontinuous, the patch size should be appropriately enlarged. Here we defined patch size as $L=2*m_{min}=32$. An overly large L will increase the computational burden and the false detection rate, while a small L will result in an excessive number of detection results. We set sliding stride S=L/2=16 to ensure that line segments crossing the boundary could be fully detected.
